# Factors associated with meeting the WHO physical activity recommendations in pregnant Colombian women

**DOI:** 10.1038/s41598-022-23947-7

**Published:** 2022-11-14

**Authors:** José Francisco López-Gil, Mikel Izquierdo, Antonio García-Hermoso, Alicia M. Alonso-Martínez, David Rincón-Pabón, Marco Antonio Morales-Osorio, Robinson Ramírez-Vélez

**Affiliations:** 1 Health and Social Research Center, Universidad de Castilla-La Mancha, Cuenca, Spain; 2 Navarrabiomed, Hospital Universitario de Navarra (HUN), Universidad Pública de Navarra (UPNA), IdiSNA, Pamplona (Navarra), Spain; 3 CIBER of Frailty and Healthy Aging (CIBERFES), Instituto de Salud Carlos III, 31006 Pamplona, Spain; 4 ZIPATEFI (Zona de Investigaciones de Posgrados, Terapia Respiratoria y Fisioterapia de Areandina), Fundación Universitaria del Área Andina, 110231 Pereira, Colombia; 5 Grupo Internacional de Investigación Neuro-Conductual-Giinco. CUC, 080014 Barranquilla, Colombia; 6 Facultad de Ciencias de la Educación, Unidad Central del Valle del Cauca (UCEVA), Túlua, Colombia; 7 Facultad de Salud, Carrera de Kinesiología, Universidad Santo Tomás, Arica, Chile; 8 Facultad de Ciencias de la Salud, Carrera de Kinesiología, Universidad Arturo Prat, Iquique, Chile

**Keywords:** Health care, Risk factors

## Abstract

In the absence of medical contraindications, physical activity (PA) can offer health maternal and fetal health outcomes during pregnancy. However, most pregnant women may not consider PA to be feasible, suitable and safe. Hence, it is essential to determine the prevalence of pregnant women who meet the PA recommendations and the possible factors associated with that meeting, since it might be important from the perspective of pregnant women's health. The aim of the present study was to establish the prevalence of meeting the World Health Organization PA recommendations for Colombian pregnant women, as well as possible factors that may be associated with meeting that recommendations. A cross-sectional study including representative data from the National Nutritional Situation Survey (2015) in Colombia was performed. Data were collected in 2015–2016. From an initial sample of 1140 Colombian pregnant women, 702 participants with complete data were included in the final analysis. PA was assessed by self-reported information through the long version of the International Physical Activity Questionnaire. Several potential factors were analysed according to four levels of the socioecological model: the individual, interpersonal, organizational and community levels. The prevalence of Colombian pregnant women who met with the PA recommendations was 7.5%. Indigenous and Mestizo pregnant women showed lower probabilities of meeting the PA recommendations [Indigenous: OR 0.05, 95% CI (0.01–0.18); Mestizo: OR 0.12, 95% CI (0.06–0.22)] than Afro-Colombian participants. Additionally, participants who lived near green and safe spaces for PA were more likely to meet the PA recommendations [OR 2.30, 95% CI (1.06–4.79)] than those who did not live near green areas. In conclusion, a low percentage of Colombian pregnant women met the new PA recommendations. The associations found according to race/ethnicity and living near green and safe areas underline the presence of fundamental disparities associated with meeting PA recommendations.

## Introduction

Physical activity (PA), which is described as all body movement generated by the contraction of muscular tissue at all phases of life, preserves and increases cardiorespiratory fitness, decreases obesity risk and its related comorbidities and leads to increased longevity^1^. These benefits have been also reported for pregnant women^2^. In the absence of medical contraindications, PA can offer health maternal and fetal health outcomes during pregnancy^2–5^. PA reduces the risk of preeclampsia, gestational diabetes and hypertension, increased body weight during pregnancy, difficulties in childbirth, postpartum depression^4^ and neonatal complications^2,5^. However, most pregnant women may not consider PA to be feasible, suitable and safe^6^. It has been recommended that those pregnant women who before pregnancy usually were physically active can continue engaging in PA during pregnancy and the postpartum period^2^. Conversely, pregnant women with an unhealthy lifestyle should be strongly encouraged to become aware of the pre-pregnancy and pregnancy phases as a feasible setting for healthier behaviours, as healthier behaviours can be adopted during the gestational period and can exert both short- and long-term benefits during pregnancy^7,8^. Despite these benefits, there are other studies showing an association between vigorous exercise and adverse outcomes (e.g., greater risk of low birthweight^9^). However, the new World Health Organization (WHO) guidelines suggest that PA has no harmful influences on birthweight or a higher risk of miscarriage^2^.

According to the most recent PA recommendations published by the WHO, all pregnant women (with the exception of those with medical contraindications) should (a) perform a minimum of 150 min of moderate intensity PA per week; (b) include a range of both muscle strengthening and aerobic activities; and (c) limit the time spent performing sedentary behaviours, replacing sedentary behaviours with active behaviours of any intensity (even light intensity), since it may also offer benefits for their health^2^. Similarly, the new WHO guidelines suggest that some PA is better than none, denoting that there are also benefits for performing moderate PA below the 150-min cut-off point compared to not performing any PA^2^. However, a decrease in PA levels is commonly observed in women during pregnancy^3,4^. This reduction is often the result of traditional beliefs about the risks of practicing PA during pregnancy. Thus, previous studies conducted in countries other than Colombia have reported a proportion of pregnant women with sufficient PA levels ranging from 20 to 32.5%^4–9^.

On the other hand, previous studies have pointed out the factors linked with engagement in PA among pregnant women, such as race/ethnicity^10–13^, socioeconomic status^8,11,12^, or educational level^8,9^. For instance, a systematic review by Gaston and Cramp^12^ pointed out that Hispanic and Asian women were most likely to not achieve the minimum amount of PA recommended. Other studies have found that pregnant women with high socioeconomic status were more likely to be active than women with low socioeconomic status^8,12^. In addition, it has been reported that greater educational level (e.g., having completed high school, college or university) is related to higher PA^14–20^. However, the factors associated with meeting PA recommendations in Colombian pregnant women have yet fully explored.

Based on the above, it seems essential to determine the prevalence of pregnant women who meet the PA recommendations and the possible factors associated with meeting the recommendations from the perspective of pregnant women's health. This analysis could offer necessary data for the establishment of both prevention and intervention programs. In addition, studies evaluating pregnant women and whether they meet the new WHO PA recommendations are scarce. Thus, the aim of this study was to establish the prevalence of meeting the new WHO PA recommendations among Colombian pregnant women, as well as the possible factors that may be associated with meeting those recommendations.

## Materials and methods

### Study design and participants

This cross-sectional study included representative data from the last available National Nutritional Situation Survey in Colombia (in Spanish, *Encuesta Nacional de Situación Nutricional* ENSIN-2015)^21^. Data were collected in 2015–2016. Briefly, this survey tried to describe the food and nutritional situations of the Colombian population within the framework of the model of social determinants as an input for the formulation, follow-up and reorientation of public policies on food and nutritional security in Colombia.

The survey was applied directly, with the interviewer visiting each of the households selected from the sample and performing a face-to-face interview with the selected person (using standardised questionnaires). This survey included a probabilistic multistage, stratified, cluster sampling strategy. Municipalities were stratified and randomised in relation to urban and rural regions. In the next phases, blocks were stratified to select the unit of analysis (i.e., households). The ENSIN-2015 included 44,202 households in 295 municipalities of the country representing 4739 groups from 177 strata by stratified multistage sampling. The ENSIN-2015 included 151,343 people, of whom 1140 were pregnant women. Among these 1140 pregnant women, 438 were excluded because of unavailable data about moderate or vigorous PA (with medical recommendations to avoid PA) or missing data and 702 were included in the final analysis (Supplementary Fig. S1).

Data from the ENSIN-2015 are public, anonymous and accessible to everyone through a well-founded application to the Colombian Ministry of Health. The ENSIN survey was approved by the PROFAMILIA Ethics Committee before data collection (ID8430/1993 Colombian Ministry of Health). The *Fundación Universitaria del Área Andina*, Pereira (ID protocol CVI2019-ZI-P33) approved the study protocol for secondary analysis by the Human Subjects Committee according to the principles of the Declaration of Helsinki of the World Medical Association. Furthermore, all participants involved were required to sign a written informed consent form before participating in this study.

### Procedures

Incomplete questionnaires for any scale were excluded to reduce the risk of bias in the analysis. The pregnancy phase was determined considering the week of the last menstrual period^22^. Then, this variable was categorised as 1st trimester (less than 14 weeks), 2nd trimester (from 14 to 26 weeks) and 3rd trimester (27 or more weeks). Likewise, participants were asked about the number of pregnancies in their lifetime.

PA in the Colombian adult population (including pregnant women) was assessed by self-reported information through the long version of the International Physical Activity Questionnaire (IPAQ)^23^. Data on the reliability/validity of the IPAQ have been indicated in a previous study performed in twelve countries^23^, where it was found that the results are comparable internationally. Prior to the application of the IPAQ, the pregnant women were asked if they had been identified as having any contraindications to PA practice by their doctor. Transport and leisure time domains of the long version of the IPAQ were assessed. Transport domain included questions related to the way of transportation from place to place (e.g., work, stores, movies). Leisure time domain involved questions related physical activities (e.g., recreation, sport, exercise or leisure). Total minutes spent performing PA in each domain were computed, and subsequently, a dichotomic variable was generated to specify meeting the PA recommendations. For this purpose, a total of 150 min of moderate activity per week was used as a cut-off point, following the new WHO recommendations^2^. The measurement instruments used were in Spanish and demonstrated adequate psychometric properties^23^.

Several potential factors were analysed following the four levels of the socioecological model offered by McLeroy et al.^24^: (1) Individual-level factors: (age, race/ethnicity, pregnancy phase and excess weight). Age was self-reported. Concerning race/ethnicity, participants choose one of the following six ethnic groups: (1) Indigenous, (2) *Rom*, (3) *Raizal del Archipiélago de San Andrés y Providencia*, (4) *Palanquero de San Basilio*, (5) Afro-Colombian and (6) none of the above. We focused on the three largest ethnic groups in Colombia (i.e., Afro-Colombian, Indigenous and Mestizo)^25,26^. Pregnancy phase that was defined as follows: 1st trimester, 2nd trimester or 3rd trimester. Excess weight that was based on the cut- off described by Atalah et al.^27^ for pregnant women. To establish this cut-off point, a reference table was theoretically defined based on the body mass index, from the 10th to the 42nd week of gestation. In the first weeks of pregnancy, the nutritional classification recommended by the WHO for non-pregnant women was used: underweight < 20.0; normal weight 20.0–24.9; overweight 25.0–29.9; and obesity ≥ 30.0. The average cumulative weight gain in the first 10 weeks of gestation was estimated to be 600 g. The "ideal" weight gain for normal women was estimated to be 20% of the initial weight. (2) Interpersonal-level factors (educational level [professional degree or higher; some university; completed high school; some high school; completed primary; or some elementary or less], socioeconomic level by the *quartile of wealth* [level I, II, III or IV]) and marital status [married, separated/divorced, widowed, single or living with a partner]. For further analyses, we recoded marital status as: “married” or “not married” (separated/divorced, widowed, single and living with a partner). (3) Organizational-level factors (type of household [nuclear family, extended family or blended/single-parent family] and television in the bedroom). (4) Community-level factors (area of residence [rural or urban], green areas [no, yes (not safe for PA) or yes (safe for PA)], health care system [contributory (including people who were affiliated with the General Social Security Health Care System by paying a fee, directly financed by the affiliated members themselves or jointly with their employers), special subsidised (including people who could not afford to pay any fees and who were affiliated with the General Social Security Health Care System through a per capita Payment Unit, totally or partially subsidised by the Colombian Government) or non-affiliated (including people temporarily linked to the General Social Security Health Care System who could not afford to pay any fees and who belonged to strata 1 or 2 while waiting to be accepted as fully affiliated members of the Subsidised Plan)]. For additional analyses, we recategorized type of the household as: “nuclear family” or “not nuclear family” (extended family and blended/single-parent family). Similarly, healthy was recoded into: “contributory” and “not contributory” (special subsidises and non-affiliated). Procedures to categorize the different factors included among the sample of Colombian pregnant women analysed are shown in Supplementary Table S1.

### Statistical analysis

Descriptive data are shown as numbers and frequencies (for categorical variables) and means and standard deviations (for continuous variables). The chi-square test was applied to verify differences between categorical variables according to the meeting of the WHO PA recommendations. Fisher’s exact test, instead of the chi-square test, was applied in case of small values of the expected cells. Conversely, differences between continuous variables by meeting WHO PA recommendations were evaluated by Mann–Whitney’s *U* test. In addition, a multilevel mixed-effects logistic regression analysis was performed, including in the same model the different factors described above (i.e., individual-level, interpersonal-level, organizational-level and community-level factors) in addition to region-specific random effects (i.e., Eastern, Central, Atlantic, Amazon, Pacific or Bogotá). Survey functions in STATA 16.1 (StataCorp, College Station, TX, USA) were used to perform the analyses to reflect the weighting for each participant. A *p*-*value* lower than 0.05 was considered statistically significant.

## Results

Table 1 shows the characteristics of the analysed sample and the relationship between socioecological factors and meeting the WHO PA recommendations. Overall, considering transport and leisure time domains, the prevalence of Colombian pregnant women who met the PA recommendations was 7.5%. According to the pregnancy phase, the prevalence was 5.2% (1st trimester), 9.3% (2nd trimester) and 6.5% (3rd trimester) (data not shown). Most of the Colombian pregnant women were Mestizo (77.5%). A higher percentage of pregnant women were from urban areas (72.9%). Similarly, most of the participants lived with a partner (not married) (61.4%). Comparisons between the included/excluded participants are shown in Supplementary Table S2 with significant differences found in relation to age and region (*p* < 0.05 for all).

**Table 1:** Socioecological correlates of the Colombian pregnant women and according to meeting the physical activity recommendations.

Variables	Total sample N = 702 100.0%	Non-meeting PA recommendations n = 649 92.5%	Meeting PA recommendations n = 53 7.5%	*p*
Age^†^	25.0 (21.0–30.0)	25.0 (21.0–30.0)	25.0 (21.0–28.0)	0.905
**Race/Ethnicity**^**‡**^				
Afro-Colombian	62 (8.6)	62 (8.6)	8 (15.1)	0.246
Indigenous	96 (13.3)	96 (13.3)	7 (13.3)	
Mestizo	544 (77.5)	544 (77.5)	38 (71.7)	
**Region**^**‡**^				
Atlantic	242 (34.5)	230 (35.4)	12 (22.6)	0.130
Eastern	84 (12.0)	78 (12.0)	6 (11.3)	
Amazon	111 (15.8)	101 (15.6)	10 (18.9)	
Bogotá	40 (5.7)	33 (5.1)	7 (13.2)	
Central	145 (20.7)	134 (20.6)	11 (20.8)	
Pacific	80 (11.4)	73 (11.2)	7 (13.2)	
**Marital status**^**‡**^				
Married	99 (14.1)	92 (14.2)	7 (13.2)	0.846
Not married	603 (85.9)	557 (85.8)	46 (86.8)	
**Educational level**^**‡**^				
Incomplete elementary or less	177 (25.2)	166 (25.6)	166 (20.8)	0.515
Complete primary or incomplete high school	213 (30.3)	198 (30.5)	198 (28.3)	
Complete high school or incomplete university	283 (40.3)	260 (40.1)	260 (43.4)	
Professional degree or higher	29 (4.1)	25 (3.9)	4 (7.5)	
**Socioeconomic status by quartile of wealth**^**‡**^				
Level I—the poorest	352 (50.1)	327 (50.4)	25 (47.2)	0.473
Level II	196 (27.9)	184 (28.4)	12 (22.6)	
Level III	112 (16.0)	100 (15.4)	12 (22.6)	
Level IV—the richest	42 (6.0)	38 (5.9)	4 (7.5)	
**Type of household **^**‡**^				
Nuclear family	345 (49.1)	319 (49.2)	26 (49.1)	0.989
Not nuclear family	357 (50.9)	330 (50.8)	27 (50.9)	
**Health care services**^**‡**^				
Contributory	223 (31.7)	203 (31.3)	20 (37.7)	0.332
Not contributory	479 (65.2)	446 (68.7)	33 (62.3)	
**Area of residence**^**‡**^				
Urban	512 (72.9)	471 (72.6)	41 (77.4)	0.451
Rural	190 (27.1)	178 (27.4)	12 (22.6)	
TV in bedroom^‡^, yes	328 (46.7)	305 (47.0)	23 (43.4)	0.614
**Green areas**^**‡**^				
No green areas	321 (45.7)	302 (46.5)	19 (35.8)	0.237
Yes (not safe for PA)	103 (14.7)	92 (14.2)	11 (20.8)	
Yes (safe for PA)	278 (39.6)	255 (39.3)	23 (43.4)	
Number of pregnancies (times) ^†^	2.0 (1.0–3.0)	2.0 (1.0–3.0)	2.0 (1.0–3.0)	0.905
**Anthropometric data **^**†,‡**^				
Height (cm)^†^	155.1 (151.4–159.0)	155.1 (151.4–159.1)	156.0 (151.3–157.0)	0.033
Weight (kg)^†^	62.7 (55.2–71.6)	6.7 (55.2–71.6)	58.4 (52.1–65.7)	0.465
BMI (kg/m^2^)^†^	26.0 (22.9–29.4)	26.1 (23.0–29.3)	25.5 (23.1–29.2)	0.568
Normal weight^a,‡^	409 (58.3)	378 (58.2)	31 (58.5)	0.972
Overweight/Obesity^a,‡^	293 (41.7)	271 (41.8)	22 (41.5)	
Pregnancy phase^b,‡^				
1st trimester	134 (19.1)	127 (19.6)	7 (13.1)	0.239
2nd trimester	322 (45.9)	292 (45.0)	30 (56.6)	
3rd trimester	246 (35.0)	230 (35.4)	16 (30.2)	

BMI: Body mass index; PA: Physical activity.

^†^Data expressed as median (interquartile range).

^‡^Data expressed as number (percentage).

^a^Prevalence of excess of weight according to the cut-off points proposed by Atalah et al.^27^ for Colombian pregnant women.

^b^According to the week of the last menstruation period.

^c^ It includes the sum of weekly minutes of moderate physical activity, vigorous physical activity and active commuting.

Table 2 indicates the results of the multilevel mixed-effect logistic regression analysis. Lower probabilities of meeting the PA recommendations were found for Indigenous [Odds Ratio [OR] = 0.05, 95% confidence interval [CI] (0.01–0.18)] or Mestizo pregnant women [OR 0.12, 95% CI (0.06–0.22)] in comparison with Afro-Colombian pregnant women. Additionally, pregnant women who lived near green areas (and safe areas for PA) [OR 2.30, 95% CI (1.06–4.79)] had greater probabilities of meeting the PA recommendations compared to those who did not live near green areas.

**Table 2:** Multilevel mixed effects logistic regression analysis with potential socioecological factors as independent variables and meeting the physical activity recommendations as a dependent variable, among Colombian pregnant women.

Predictors	OR^a^	95% CI	*p*
Age (per one year)	1.02	0.95–1.09	0.597
**Race/Ethnicity**			
Afro-Colombian	1		
Indigenous	**0.05**	**0.01–0.18**	** < 0.001**
Mestizo	**0.12**	**0.06–0.22**	** < 0.001**
**Marital status**			
Married	1		
Not married	0.76	0.41–1.49	0.384
**Educational level**			
Incomplete elementary or less	1		
Complete primary or incomplete high school	0.55	0.29–1.06	0.073
Complete high school or incomplete university	0.67	0.24–1.84	0.435
Professional degree or higher	0.60	0.06–6.05	0.667
**Socioeconomic status by quartile of wealth**			
Level I—the poorest	1		
Level II	0.79	0.16–3.99	0.777
Level III	0.84	0.22–3.24	0.797
Level IV—the richest	0.82	0.13–5.32	0.832
**Type of household**			
Nuclear family	1		
Not nuclear family	1.69	0.68–4.21	0.263
**Health care services**			
Contributory	1		
Not contributory	0.65	0.26–1.59	0.344
**Area of residence**			
Rural	1		
Urban	1.42	0.23–8.90	0.710
TV in bedroom (yes)	0.55	0.21–1.46	0.231
**Green areas**			
No green areas	1		
Yes (not safe for PA)	1.22	0.48–3.11	0.671
Yes (safe for PA)	**2.30**	**1.06–4.79**	**0.035**
Number of pregnancies (per one pregnancy)	0.86	0.59–1.25	0.440
Excess weight (yes)^‡^	0.64	0.25–1.61	0.341
**Pregnancy phase**^**§**^			
1st trimester	1		
2nd trimester	2.02	0.70–5.78	0.191
3rd trimester	0.63	0.21–1.84	0.395

Data expressed as a odds ratio and 95% confident intervals.

Bold indicates correlates with *p*-value < 0.05.

*OR* odds ratio, *PA* physical activity.

^†^Non-meeting the physical activity recommendations selected as a reference group.

^‡^Prevalence of excess of weight according to the cut-off points proposed by Atalah et al.^27^ for pregnant women.

^§^According to the week of the last menstruation period.

## Discussion

Our results showed that the prevalence of meeting the new WHO PA recommendations among Colombian pregnant women was 7.5%. The probabilities of meeting the recommendations were lower in Indigenous and Mestizo participants compared to Afro-Colombian participants. Furthermore, participants who lived near green areas and safe for PA were more likely to meet the PA recommendations than those who did not live near green areas or lived near green areas but unsafe for PA.

Considering that the prevalence of meeting with the PA recommendations among Colombian women is 42.7%, this finding highlights the importance of promoting PA levels among Colombian pregnant women. Our report is consistent with that by Sánchez-Martínez et al. who also showed that the prevalence of physical activity in pregnant women was 12.5%, 28.6%, and 36.3% in the leisure-time, commuting and global domains, respectively^28^. Similarly, the prevalence of Colombian pregnant women who met the PA recommendations was lower than that in other studies from other countries^4–9^. Our results are not in line with those reported by Hailemariam et al.^11^ in Ethiopia, Davenport et al.^3^ in the USA and Baena-García in Spain^29^. In these cross-sectional studies, the authors reported a prevalence of 20.7%, 21.0% and 25.0% of pregnant women who engaged in PA for a minimum of 150 min weekly, respectively^28^. Another longitudinal study carried out in Italy showed that the prevalence of pregnant women who met the PA recommendations varied throughout pregnancy: 26% (1st trimester), 45% (2nd trimester) and 38% (3rd trimester) in the normal weight group and 20%, 23% and 15% in the overweight/obesity group, respectively^30^. Additionally, Collins et al.^31^, in their prospective birth cohort study among white British women and women of Pakistani origin, found that 61% and 26%, respectively, achieved the minimum amount of PA recommended. Notwithstanding, our results are in line with those of one cross-sectional study performed among Brazilian pregnant women that showed that 7.2% (1st trimester), 7.6% (2nd trimester) and 4.7% (3rd trimester) did not meet the PA recommendations^7^. One possible reason that could, at least partially, justify this low prevalence of pregnant women meeting the PA recommendations is due to the different methods applied to assess PA levels, such as different questionnaires (e.g., the Pregnancy Physical Activity Questionnaire, the Paffenbarger Physical Activity Survey) or more objective measures (e.g., accelerometers). Moreover, the different locations and cultures among these studies could also explain the discrepancies. Additionally, it must be considered that the new WHO recommendations do not consider low-intensity PA as meeting the recommendations, and the mentioned studies reporting low levels of PA usually exclude periods with a duration less than 10 min from the global PA computation. This procedure has been disapproved, since it could diminish the total sum of estimated moderate-to-vigorous PA^17,33,34^, and is contrary to the recent WHO slogan “every move counts”^2^. One example of this consideration is the study performed by Mendinueta et al.^16^, who indicated that when they applied this procedure, the prevalence of participants meeting the recommendations clearly changed from 77 to 30% (women in the 1st trimester) and from 85 to 32% (women in the 2nd trimester).

Regarding the potential factors associated with meeting the WHO PA recommendations, we found lower probabilities of meeting these recommendations in Indigenous and Mestizo participants compared to Afro-Colombian participants. This finding agrees with some previous studies^13,32–35^, but is not in line with others^11,12^ which found that black pregnant women had lower PA levels. However, caution is required when comparing these studies. The different tools applied to measure PA levels, the different geographic locations, the cultural differences within participants of the same race/ethnicity depending on the location, or even the different age phases of the participants might affect the results obtained. Likewise, it has been recommended that formative data related to PA questions be collected to obtain a more appropriate equivalence of the items when applied to a different cultural group. All these aspects could (at least partially) explain the discrepancies.

According to educational level, we found no association between educational level and meeting the PA recommendations. In this sense, the results of Mendinueta et al.^16^ showed that a lower educational level was linked to higher PA, contrary to the results of Nascimento et al.^7^, as well as those by Todorovic et al.^15^. However, Mendinueta et al.^16^ authors measured PA levels objectively, which could explain the discrepancies. In relation to socioeconomic status, the findings of Nascimento et al.^36^ advised that high socioeconomic status and receiving prenatal care through private services were associated with greater PA, which was contrary to our findings. The low prevalence of Colombian pregnant women who met the PA recommendations could, at least partially, explain the lack of association between these variables. Similarly, we must consider that this is one of the first studies assessing PA recommendations on the basis of the newest PA recommendations indicated by the WHO^2^, and most of the studies analysed included the time of active commuting in the computation of PA levels. Notwithstanding, we should acknowledge that these PA guidelines have a long-standing history in other organizations/countries (e.g., The American College of Obstetricians and Gynecologists, The American College of Sports Medicine, The Canadian Physical Activity Guidelines, etc.).

Importantly, our findings indicate that living near green and safe areas for PA was associated with higher probabilities of meeting the PA recommendations than not living near green areas. Supporting our results, one study performed in England indicated that participants living in the greenest quintile showed higher odds of engaging in five sessions of moderate or vigorous PA for a duration of at least 30 min each week than those living in the lowest green quintile^37^. Conversely, another study conducted in New Zealand showed no relationship between exposure to green areas and PA engagement. Notwithstanding, as these authors indicated, the lack of an association does not mean (at least necessarily) that pregnant women living in green spaces will not have better health during pregnancy^38^. Thus, the different criteria when evaluating the distance to green areas, as well as the different geographical locations, could justify the discrepancies in the results.

This study was no without limitations. First, because of the cross-sectional design of this study cause and effect relationship cannot be established. Future studies are required to report the best separation of daily movement behaviours for an adequate health status during pregnancy, especially longitudinal and intervention studies. Second, the associations between the factors and the outcome (i.e., meeting the PA recommendations) could be distorted by the other variables that were not analysed (i.e., confounding bias). However, we included several potential factors in our analysis to address this limitation. Third, the reliability of self-reported PA information, which might include systematic bias (e.g., social desirability, recall bias) and lead to underestimation or overestimation. In addition, the provision of misleading information was possibly reduced because the questionnaires were completed anonymously. Furthermore, to avoid self-report bias we used the IPAQ, which is a valid and reliable questionnaire that provides data on PA dosage (frequency, type, intensity, duration) and has been recommended for surveillance and studies designed to report PA information in Latin America (especially transport and leisure time sections)^23^. Despite this fact and due to the higher precision in measuring PA, the use of more objective measures (e.g., pedometers, accelerometers) instead of self-reported measures (e.g., the IPAQ) has been suggested^39^. Unfortunately, the use of this combination is not yet feasible in epidemiological studies. Fourth, we found statistical differences between excluded/included participants which could introduce some selection bias. However, these differences were small and did not appear to be clinically relevant. Likewise, no significant differences were found for our main outcome (i.e., meeting the PA guidelines). Regarding the strengths of this study, this is, to our knowledge, one of the first studies assessing individuals meeting the PA recommendations by applying the newest WHO criteria^2^. Additionally, our study included a large national sample of pregnant women from a low-middle-income country (i.e., Colombia) and, to date, this study is one of the first to establish the percentage of Colombian pregnant women meeting the PA guidelines. Moreover, we used a socioecological model to analyse the possible factors that could affect meeting the recommendations. This choice was based on providing a better understanding of how some factors could be related to meeting health guidelines.

## Conclusion

Despite the benefits of PA during pregnancy, most of the Colombian pregnant women analysed are not sufficiently active. These low PA levels could severely affect both their health and their child’s health^3^. The associations found according to race/ethnicity and living near green and safe areas for PA underline the presence of fundamental individual, interpersonal and community disparities for meeting PA recommendations among Colombian pregnant women.

## Supplementary Information

Supplementary Figure S1.

Supplementary Table S1.

Supplementary Table S2.

## Data Availability

The datasets generated during and/or analysed during the current study are available from the corresponding author on reasonable request.
