# Supplementary figures and images for: Factors associated with meeting the WHO physical activity recommendations in pregnant Colombian women
Source: Sci Rep. 2022 Nov 14;12:19500. doi: 10.1038/s41598-022-23947-7 (PMC9663497; doi:10.1038/s41598-022-23947-7)

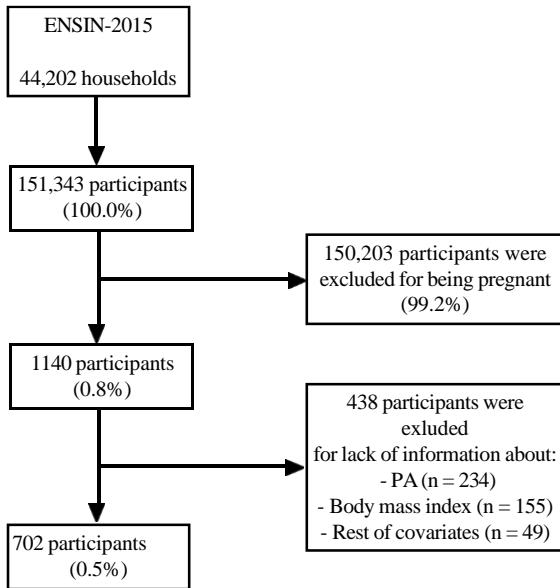

**Fig S1.** Flowchart depicting final study-subject selection from the ENSIN-2015 population

Supplement: Supplementary file 1 — Supplementary Figure S1. [file 41598_2022_23947_MOESM1_ESM.pdf]
