# Supplementary material for: Factors associated with meeting the WHO physical activity recommendations in pregnant Colombian women
Source: Sci Rep. 2022 Nov 14;12:19500. doi: 10.1038/s41598-022-23947-7 (PMC9663497; doi:10.1038/s41598-022-23947-7)
Supplement: Supplementary file 2 — Supplementary Table S1. [file 41598_2022_23947_MOESM2_ESM.docx]

**Table S1.** Procedures to categorize the different correlates included among the sample of Colombian pregnant women analysed.

| **Factor** | **Method** | **Categorization** |
| --- | --- | --- |
| Age | Single question: “What is your age?” | Continuous variable. |
| Race/Ethnicity | Single question: “What is your race/ethnicity?” | Re-coded as: (1) “Afro-Colombian”; (2) “Indigenous”; or (3) “Mestizo”. |
| Marital status | Single question: “What is your current marital status?” | Original categories: (1) “Married”; (2) “Separated/divorced”; (3) “Widow”; (4) “Single”; or (5) “Living with partner”.  Re-coded as: (1) “Married”; (2) “Not married” (“Separated/divorced”, “Widow”, “Single”, and “Living with partner”). |
| Educational level | Single question: “What is your educational level?” | Re-coded as: (1) “Incomplete elementary or less”; (2) “Complete primary or incomplete high school”; (3) “Complete high school or incomplete university”; (4) or “Professional degree or higher”. |
| Socioeconomic status | The SISBEN is an instrument for identifying the poor and vulnerable population used by the State to allocate social spending, through a database consolidated by the *National Planning Department* of Colombia. Quartile of wealth is constructed based on the information obtained through the application of a questionnaire to households (socioeconomic characterization form), through which the characteristics of housing, habitability and other socioeconomic conditions and quality of life of households and each of its members are known. | Re-coded as: (1) “Level I”; (2) “Level II”; (3) “Level III”; or (4) “Level IV”. |
| Type of household | Single question: “Which distribution by type of family structure is most similar to yours?” | Original categories: (1) “Nuclear family”; (2); “Extended family”; (3) “Blended/single-parent”.  Re-coded as: (1) “Nuclear family”; (2); “Not nuclear family” (“Extended family” and “Blended/single-parent”). |
| Health care services | Single question: “What is your type of health insurance?” | Original categories: (1) “Contributory”, (2) “Special subsidised”; or (3) “Non-affiliated”.  Re-coded as: (1) “Contributory”, (2) “Not contributory” (“Special subsidised” and “Non-affiliated”). |
| Area of residence | Area of residence was categorized as urban or rural area. Urban area is characterized by being made up of groups of contiguous buildings and structures grouped in blocks, which are mainly delimited by streets, races or avenues. Generally, it is provided with essential services such as water, sewage, electricity, hospitals and schools, among others. Rural area is characterized by the dispersed arrangement of housing and agricultural and livestock farms. It does not have a layout or nomenclature of streets, highways, avenues, and others. In general, it does not have public services and other types of facilities typical of urban areas. | Re-coded as: (1) “Urban”; or (2) “Rural”. |
| TV in bedroom | Single question: “Do you have a TV in your room?” | Re-coded as: (0) “No”; or (1) “Yes”. |
| Green areas + Safe place for PA | Single question: “Are there any parks or green areas in your neighborhood?”  Single question: “Is that park safe?” | Re-coded as: (0) “No”; (1) Yes (not safe for PA); or (2) “Yes (safe for PA)”. |
| Number of pregnancies | Single question: “How many pregnancies during your life?” | Continuous variable. |
| Excess weight | BMI was determined by dividing weight (kg) and height (m). Excess weight was established according to the cut-off points proposed by Atalah et al. (Atalah et al., 1997) | Re-coded as: (0) “No excess weight”; or (1) “Excess weight”. |
| Pregnancy phase | Pregnancy phase was determined considering the week of the last menstrual period (Gómez-Sánchez et al., 2013). Then, this variable was categorized as 1^st^ trimester (<14 weeks), 2^nd^ trimester (14-26 weeks), and 3^rd^ trimester (≥27 weeks). Likewise, participants were asked about the number of pregnancies in their lifetime. | Re-coded as: (1) “1^st^ trimester”; (2) “2^nd^ trimester”; or (3) “3^rd^ trimester”. |
| PA Recommendations | IPAQ was used to estimate the min of PA engaged in four different domains. The WHO cut-off point of 150 minutes (or higher) for PA was applied (Bull et al., 2020). | Re-coded as: (0) “Non-meeting PA”; or (1) “Meeting PA”. |

BMI, body mass index; IPAQ, International Physical Activity Questionnaire; PA, Physical activity; SISBEN, *Sistema de Identificación de Potenciales Beneficiarios de Programas Sociales*.
