# Supplementary material for: Factors associated with meeting the WHO physical activity recommendations in pregnant Colombian women
Source: Sci Rep. 2022 Nov 14;12:19500. doi: 10.1038/s41598-022-23947-7 (PMC9663497; doi:10.1038/s41598-022-23947-7)
Supplement: Supplementary file 3 — Supplementary Table S2. [file 41598_2022_23947_MOESM3_ESM.docx]

**Table S2.** Comparisons between included/excluded Colombian pregnant women.

| Variables | Total sample (N) | Excluded participants | Analyzed sample  n = 702 | *p* |
| --- | --- | --- | --- | --- |
| Age ^†^ | 1140 | 26.6 (6.3) | 25.7 (5.7) | 0.022 |
| Race/Ethnicity ^‡^ |  |  |  |  |
| Afro-Colombian | 111 | 49 (11.7) | 62 (8.6) | 0.276 |
| Indigenous | 148 | 52 (12.4) | 96 (13.3) |  |
| Mestizo | 862 | 318 (75.9) | 544 (77.5) |  |
| Region ^‡^ |  |  |  |  |
| Atlantic | 364 | 122 (27.9) | 242 (34.5) | 0.046 |
| Eastern | 139 | 55 (12.6) | 84 (12.0) |  |
| Amazon | 184 | 73 (16.7) | 111 (15.8) |  |
| Bogotá | 61 | 21 (4.8) | 40 (5.7) |  |
| Central | 236 | 91 (20.8) | 145 (20.7) |  |
| Pacific | 156 | 76 (17.4) | 80 (11.4) |  |
| Marital status ^‡^ |  |  |  |  |
| Married | 168 | 69 (15.8) | 99 (14.1) | 0.707 |
| Separated / Divorced | 68 | 23 (5.3) | 45 (6.4) |  |
| Widow | 3 | 2 (0.5) | 1 (0.1) |  |
| Single | 205 | 79 (18.0) | 126 (17.9) |  |
| Living with a partner | 696 | 265 (60.5) | 431 (61.4) |  |
| Educational level ^‡^ |  |  |  |  |
| Incomplete elementary or less | 281 | 104 (24.5) | 177 (25.2) | 0.296 |
| Complete primary or incomplete high school | 349 | 136 (32.1) | 213 (30.3) |  |
| Complete high school or incomplete university | 440 | 157 (37.0) | 283 (40.3) |  |
| Professional degree or higher | 56 | 27 (6.4) | 29 (4.1) |  |
| Socioeconomic status by quartile of wealth ^‡^ |  |  |  |  |
| Level I – the poorest | 606 | 254 (58.0) | 352 (50.1) | 0.069 |
| Level II | 294 | 98 (22.4) | 196 (27.9) |  |
| Level III | 173 | 61 (13.9) | 112 (16.0) |  |
| Level IV – the richest | 67 | 25 (5.7) | 42 (6.0) |  |
| Type of household ^‡^ |  |  |  |  |
| Nuclear family | 586 | 241 (56.4) | 345 (49.1) | 0.057 |
| Extended family | 527 | 180 (42.2) | 347 (49.4) |  |
| Blended or single-parent family | 16 | 6 (1.4) | 10 (1.4) |  |
| Health care services ^‡^ |  |  |  |  |
| Contributory or special | 373 | 150 (34.7) | 229 (31.7) | 0.570 |
| Subsidized | 726 | 269 (62.3) | 471 (65.2) |  |
| Non-affiliated | 35 | 13 (3.0) | 22 (3.0) |  |
| Area of residence ^‡^ |  |  |  |  |
| Urban | 834 | 322 (73.5) | 512 (72.9) | 0.829 |
| Rural | 306 | 116 (26.5) | 190 (27.1) |  |
| TV in bedroom ^‡^, yes | 414 | 86 (39.6) | 328 (46.7) | 0.009 |
| Green areas ^‡^ |  |  |  |  |
| No green areas | 418 | 19 (35.8) | 321 (45.7) | 0.900 |
| Yes (not safe for PA) | 132 | 11 (20.8) | 103 (14.7) |  |
| Yes (safe for PA) | 356 | 23 (43.4) | 278 (39.6) |  |
| Number of pregnancies (times) ^†^ | 1130 | 2.4 (1.9) | 2.3 (1.5) | 0.466 |
| Anthropometric data ^†,‡^ |  |  |  |  |
| BMI (kg/m^2^) ^†^ | 955 | 26.1 (6.1) | 26.1 (6.4) | 0.820 |
| Normal weight ^b,‡^ | 555 | 146 (57.7) | 409 (58.3) | 0.878 |
| Overweight/Obesity ^b,‡^ | 400 | 107 (42.3) | 293 (41.7) |  |
| Pregnancy phase ^b, ‡^ |  |  |  |  |
| 1^st^ Trimester | 187 | 53 (12.1) | 134 (19.1) | 0.485 |
| 2^nd^ Trimester | 427 | 105 (24.0) | 322 (45.9) |  |
| 3^rd^ Trimester | 341 | 95 (21.7) | 246 (35.0) |  |
| Physical activity ^†,‡^ |  |  |  |  |
| Weekly moderate PA (min) ^†^ | 906 | 16.1 (87.1) | 10.4 (69.9) | 0.341 |
| Weekly vigorous PA (min) ^†^ | 906 | 21.9 (1.4) | 2.0 (18.5) | 0.148 |
| Weekly active commuting (min) ^†^ | 906 | 239.7 (69.1) | 27.6 (94.3) | 0.664 |
| Weekly global PA (min) ^c,†^ | 906 | 40.7 (118.9) | 40.0 (128.6) | 0.948 |
| Meeting the PA recommendations (%, yes) ^‡^ | 71 | 18 (8.8) | 53 (7.5) | 0.551 |

^a^ Prevalence of excess of weight according to the cut-off points proposed by Atalah et al. (1997) for Colombian pregnant women. ^b^ According to the week of the last menstruation period. ^c^ It includes the sum of weekly minutes of moderate physical activity, vigorous physical activity and active commuting. ^†^ Data expressed as median (interquartile range); ^‡^ Data expressed as number (percentage). BMI: Body mass index; PA: Physical activity.
